# Quantification of the content of cannabidiol in commercially available e-liquids and studies on their thermal and photo-stability

**DOI:** 10.1038/s41598-020-60477-6

**Published:** 2020-02-28

**Authors:** Carlo Mazzetti, Emanuele Ferri, Monica Pozzi, Massimo Labra

**Affiliations:** 1 Department of Biotechnology and Biosciences, University of Milano-Bicocca, Piazza della Scienza 2, Milano, 20126 Italy Italy; 2 TRUSTICERT SRL, via Mazzini 18/C, 22036 Erba, Italy

**Keywords:** Drug delivery, Drug regulation, Therapeutics

## Abstract

Cannabidiol (CBD) has become a buzzword in many products that have bloomed on the market. The scientific community and some authorities have recently raised concerns on the quality of these products. In particular, the discrepancy between the labelled and the real content of cannabidiol in liquids for e-cigarettes seems to be emerging as a major issue. Furthermore, to-date no studies have been carried out on the chemical stability upon storage of these type of products. The aim of this work was to investigate the accuracy in labelling of thirteen commercially e-liquids containing CBD and the effects of different storage conditions on their quality. The results showed that only 38% of samples were labelled within a ±10% variance from their labels. Stability tests showed a maximum degradation of CBD when samples were stored at 37 °C for 30 days with average values up to 20%. The effect of light was lower but still significant with averages values up to 15% degradation after 30 days. In conclusion, we believe that health authorities should regulate and control this market more stringently to protect customers and their health. Furthermore, our stability tests have shown that if clear indications are provided on the best storage conditions, the quality of these products can be mostly preserved.

## Introduction

**Figure 1:**
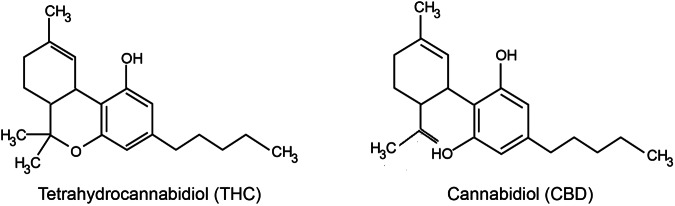
Chemical structures of THC and CBD.

The family of phytocannabinoids, produced by Cannabis sativa L, counts for 113^1^ different secondary metabolites among which tetrahydrocannabinol (THC) is the best known for its therapeutic and recreational uses^2^. Indeed, its anti-inflammatory, analgesic, appetite-stimulant and antiemetic properties^3^ have been widely studied as well as its psychotropic effects induced by the activation of CB1 and CB2 receptors in the brain^4^. Cannabidiol (CBD) is another member of this family that has only recently gained attention in the scientific community despite having been discovered decades earlier than THC^5^. This is likely due to its commercial potential that has grown over the past few years where CBD has become a buzzword in everything from skin creams to dog treats. Despite CBD shares several structural features with THC (Fig. 1), it is non-intoxicating in comparison to the latter. In particular, CBD has very low affinity for the orthosteric binding site of CB1^6–10^, but acts as a negative allosteric modulator of CB1 receptors^11^.

In 2018, the FDA approved the only therapeutic application of cannabidiol, a pure, plant-based, pharmaceutical grade extract known as Epidiolex, for the treatment of two rare and severe forms of epilepsy, Lennox-Gastaut and Dravet syndromes. In clinical trials and research studies, CBD has been administered orally as either a capsule or dissolved in an oil solution. Unfortunately, its oral bioavailability has been estimated to be 6%, due to significant first-pass metabolism^12^, which has resulted in doses ranging from 100 up to 800 mg/day^13^. According to the manufacturer, GW Pharmaceuticals, Epidiolex costs a patient, without insurance, $32,500 *per* year hence, despite the high tolerance level of CBD^14^, routes with higher bioavailability, which in turn result in lower doses, would be particularly desirable. Inhalation of phytocannabinoids have been proved to be one of the most efficient administration route in terms of bioavailability with values reaching around 30%^15^. Besides smoking cigarettes, new devices have been developed to vaporize phytocannabidiols that use electric power to heat the product to the point of vaporization. Electronic cigarettes (EC) are battery-powered portable vaporizer devices and since their first introduction in 2003 as an alternative route to traditional tobacco for nicotine delivery system, they have steadily gained popularity among adults and adolescents becoming in the latter, a more common tobacco product than conventional cigarettes^16–18^. In EC, nicotine is dissolved in a solution made of variable ratios of two chemical compounds, namely propylene glycol (PG) and vegetable glycerine (VG), and added flavours to form a liquid, known as e-liquid, which is then vaporized by a coil. Several studies have shown that PG and VG can undergo thermal degradation during vaping to form toxic and potentially toxic compounds among which formaldehyde, acetaldehyde and acrolein^19^. Their concentration is strictly related to the operative conditions of the devices and increases with increasing temperatures due to high battery output voltages applied to EC^20^. Following the recent legalization of cannabis in some U.S. states, there has been a blooming of e-liquids containing cannabinoids on the market worldwide as a new drug delivery system. In particular, several CBD products have become available with a wide range of concentrations (from 10 up to 100 mg per mL of solution) and medicinal claims. The continuous misleading labels of these products prompted the FDA in 2015 and 2016 to issue warning letters to companies marketing an unapproved drug in their products for therapeutic benefits^21^. Furthermore, two separated studies have highlighted the discrepancy between the declared labelled concentrations of cannabinoids and the real contents. Indeed, Pearce *et al*. analysed two commercial marijuana e-liquids claiming to contain 3.3 mg/mL CBD and found that the real contents were around twice as much^22^. In 2017, FDA carried out tests on the chemical content of CBD in commercially available products confirming this label inaccuracy^23^. Another aspect that needs to be addressed is the storage stability of e-liquids containing CBD. To date no studies have been published on the influence of storage conditions on the quality of these products. These lacking data are extremely important considering the chemical stability of CBD that undergoes oxidation in presence of oxygen to form mono- and dimeric hydroquinones and degradation when exposed to light^24^. Temperature could be another important factor in terms of quality of the final product to be monitored during shipping and storage. The aim of these studies was to investigate the feasibility of EC as a potential alternative CBD delivery system by gaining information on the actual state of the accuracy in labelling e-liquids containing CBD and by providing explorative data on their best storage conditions.

## Results

The results obtained for the thirteen commercially available e-liquids are shown in Table 1. Despite the label concentrations of samples ranged from 10 to 100 mg/mL, we found much lower values, from 0,8 to 35,1 mg/mL, which clearly indicates a wide discrepancy between the declared and the real concentration of CBD in these products. The real concentrations have been classified in three major groups, underestimated (<−10%), within +10% variance and overestimated (>+10%), following the USP product monographs (as compared with substance monographs) tolerance of 90–110% of the label claim for both manufactured products and compounded preparations^25^. Five samples, namely B, G, H, I and L, showed an underestimation of the concentration of CBD with values ranging between −11 and −92% of the label claim. Five samples (C, E, M, N and O) were within a +10% variance whereas three samples (A, D and F) showed an overestimation of the content of CBD with values ranging between 13, 14 and 17% respectively. These warning figures might be explained either by low manufacturing practices used by companies or by degradation of the phytocannabinoid as a result of wrong storage conditions of these products. In order to validate the latter hypothesis, we carried out stress tests on the thirteen samples upon storage.

**Table 1:** Results of the quantification of CBD by HPLC in the thirteen commercial products.

Sample	Company	Solvent PG/VG	Label CBD Concentration (mg/mL)	Experimental CBD Concentration (mg/mL)	Percent Difference (%)
A	1	40/60	20	22,6 ± 0,2	13 ± 1
B	1	40/60	10	8,9 ± 0,3	−11 ± 3
C	2	50/50	10	10,9 ± 0,4	9 ± 4
D	3	n.d.	10	11,4 ± 0,5	14 ± 1
E	4	80/20	20	18,0 ± 0,1	−10 ± 1
F	5	100/0	30	35,1 ± 0,6	17 ± 2
G	5	50/50	10	0,8 ± 0,1	−92 ± 1
H	5	50/50	25	2,2 ± 0,2	−91 ± 1
I	5	50/50	50	4,0 ± 0,5	−92 ± 1
L	6	50/50	100	8 ± 1	−92 ± 1
M	7	n.d.	25	23,5 ± 1,2	−6 ± 5
N	8	50/50	20	20,8 ± 0,2	4 ± 1
O	9	n.d.	20	20,6 ± 0,6	3 ± 3

(Percent difference is calculated as the ratio between experimental CBD concentration and label CBD concentration which is then multiply for hundred and the result subtracted of hundred).

The results of Table 2 show that samples stored at 4 °C underwent an average decrease in the concentration of CBD within 5% with the only exceptions being samples B and N with slightly higher percentages. Samples stored at RT showed a similar behaviour with the exceptions of A, B and N where the decrease in the concentration of CBD were equal to 7, 6 and 11% respectively. Storing samples at 37 °C turned out to be more stressful in terms of decrease in concentration of CBD with eight samples showing average values above 10% and five samples, C, D, M, N and O, values between 5 and 10%. Statistical analyses indicated that only samples N and O were not significantly different in their final CBD concentration when comparing the three different storage temperatures. Sample D and G showed a better CBD conservation only when stored at 4 °C and sample M was found to conserve a higher CBD concentration after 30 days at room temperature than at 37 °C, but CBD concentration did not decrease in a significant manner when comparing 4 °C and 37 °C conditions. All the other samples were found to be comparable if stored at 4 °C or at room temperature and to be affected by conservation at 37 °C.

**Table 2:** Effect of temperature on the thirteen samples upon storage. Superscripts indicate the significance in the comparisons among the three storage temperature for each sample (*p *< 0.05).

Sample	C_0_ (mg/mL)	C_F_ at 4 °C (mg/mL)	C_F_ at RT (mg/mL)	C_F_ at 37 °C (mg/mL)
A	22,6	21,40 ± 0,2^a^	21,00 ± 0,9^a^	19,2 ± 0,7^b^
B	8,9	8,40 ± 0,35^a^	8,40 ± 0,35^a^	7,30 ± 0,3^b^
C	10,9	10,60 ± 0,2^a^	10,60 ± 0,3^a^	9,80 ± 0,2^b^
D	11,4	11,40 ± 0,1^a^	11,10 ± 0,35^ab^	10,70 ± 0,2^b^
E	18,0	18,00 ± 0,2^a^	17,40 ± 0,2^a^	16,00 ± 0,4^b^
F	35,1	34,80 ± 0,3^a^	34,80 ± 0,3^a^	30,00 ± 0,7^b^
G	0,8	0,79 ± 0,01^a^	0,76 ± 0,01^ab^	0,70 ± 0,04^b^
H	2,2	2,18 ± 0,03^a^	2,08 ± 0,04^a^	1,75 ± 0,11^b^
I	4,0	3,95 ± 0,05^a^	3,80 ± 0,09^a^	3,50 ± 0,12^b^
L	8	7,90 ± 0,1^a^	7,60 ± 0,1^a^	7,00 ± 0,4^b^
M	23,5	23,00 ± 0,5^ab^	23,50 ± 0,25^a^	22,00 ± 0,7^b^
N	20,8	19,00 ± 0,6^a^	18,60 ± 0,2^a^	18,80 ± 0,4^a^
O	20,6	20,40 ± 0,2^a^	19,60 ± 0,8^a^	18,80 ± 0,8^a^

**Figure 2:**
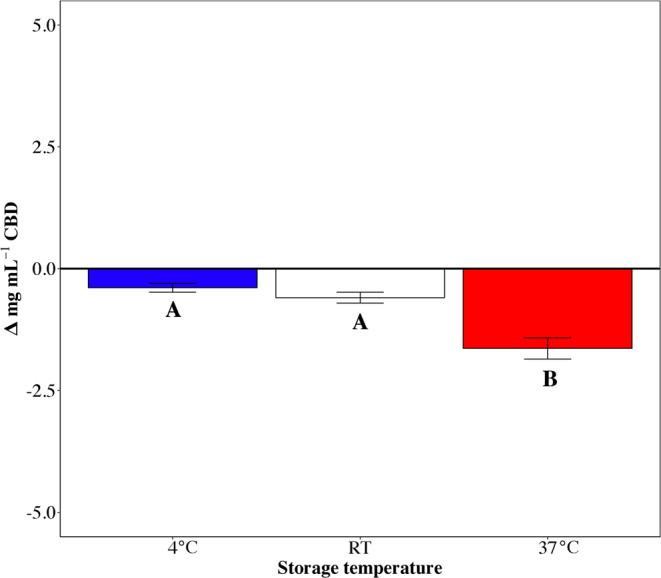
Δ_[CBD]_ represents the difference in CBD concentration (mg/ml) after 30 days at three different storage temperature conditions. Data are mean ± SEM. Superscripts indicate the significance in the comparison among the three conditions (*p* < 0.05).

Figure 2 confirms a general decrease of CBD concentration in samples stored at 37 °C with an average Δ[CBD] = −1.64 ± 0.22 mg/ml. Difference is statistically significant if compared to samples stored at both 4 °C (t = 7.9, p < 0.001) and room temperature (t = 6.6, p < 0.001) that show a Δ[CBD] = 0.39 ± 0.09 mg/ml and 0.59 ± 0.11 mg/ml respectively.

The effect of light on the thirteen samples was also investigated by storing the solutions for 30 days at RT (same samples used for the thermal stability studies) placing alongside two identical samples of 1 mL each, one wrapped in aluminium foil (dark control, Table 2 entry CF at RT), under a natural day light exposure. Results (Table 3, percent difference rt + photo) showed a net detrimental effect of light on the chemical stability of CBD with an average decrease in the CBD concentration of 13% against samples conserved in the dark which showed an average decrease of 4%. Statistical analyses indicated that samples D, E, H, M, O did not undergo to a significant CBD decrease between treatments while all the other were significantly affected in their final CBD concentration by light exposure.

**Table 3:** Effect of da light on the thirteen samples upon storage. Superscripts indicate the significance in the comparisons among the three storage temperature for each sample (*p* < 0.05).

Sample	C_0_ (mg/mL)	C_F_ RT + Photo (mg/mL)	C_F_ Dark Control (mg/mL)
A	22,6	19,8 ± 0,45^a^	21 ± 0,9^b^
B	8,9	7,0 ± 0,35^a^	8,4 ± 0,35^b^
C	10,9	9,4 ± 0,3^a^	10,6 ± 0,3^b^
D	11,4	10,3 ± 0,1^a^	11,1 ± 0,35^a^
E	18,0	15,8 ± 0,4^a^	17,4 ± 0,2^a^
F	35,1	30,3 ± 0,4^a^	34,8 ± 0,3^b^
G	0,8	0,72 ± 0,01^a^	0,76 ± 0,01^b^
H	2,2	1,8 ± 0,05^a^	2,08 ± 0,04^a^
I	4,0	3,6 ± 0,05^a^	3,80 ± 0,09^b^
L	8	7,2 ± 0,1^a^	7,6 ± 0,1^b^
M	23,5	21,0 ± 0,5^a^	23,5 ± 0,25^a^
N	20,8	17,4 ± 0,4^a^	18,6 ± 0,2^b^
O	20,6	18,8 ± 0,4^a^	19,6 ± 0,8^a^

**Figure 3:**
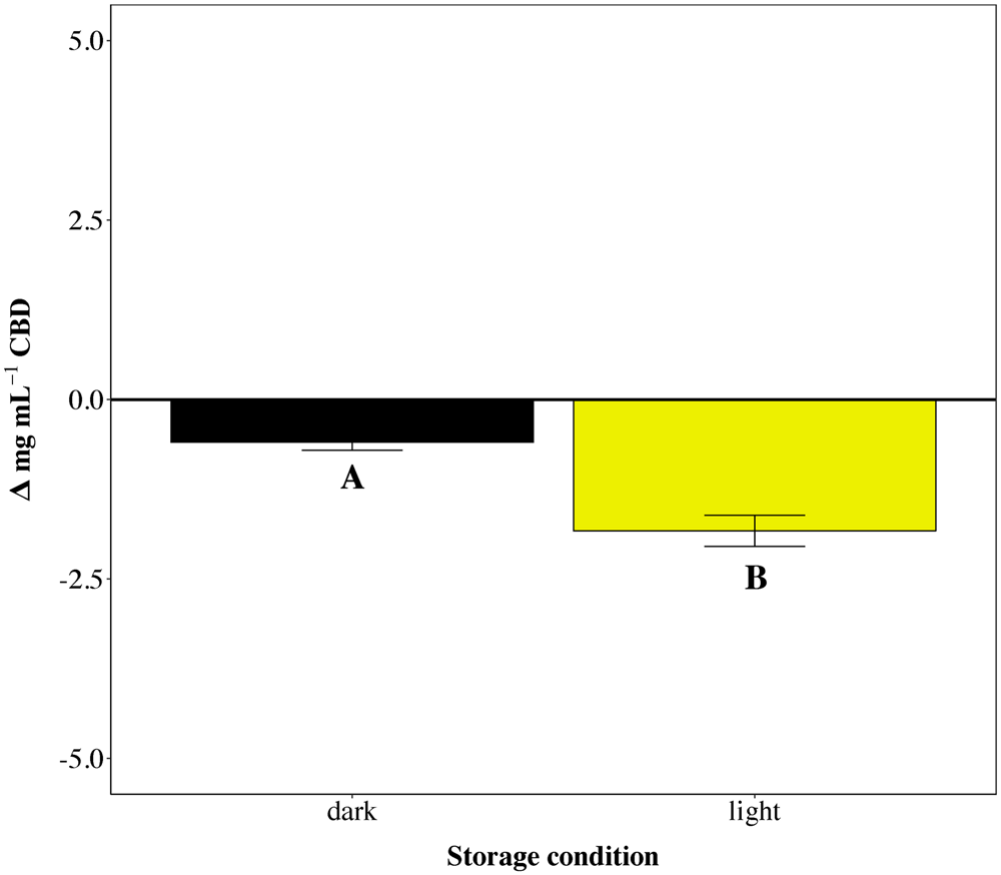
Δ_[CBD]_ represents the difference in CBD concentration (mg/ml) after 30 days in two light storage conditions. Data are mean ± SEM. Superscripts indicate the significance in the comparison among the three conditions (*p* < 0.05).

Figure 3 suggests that light exposure significantly affected CBD concentration (χ² = 43.2, p < 0.001) with a Δ[CBD] = −1.83 ± 0.22 mg/ml after 30 days of conservation exposed to light against a Δ[CBD] = −0.59 ± 0.11 mg/ml in dark storage conditions.

## Discussion

Thirteen commercially available e-liquids containing CBD were purchased online and analysed by liquid chromatography to gain information on the accuracy of the concentration of the phytocannabinoid on their labels. This has been achieved by developing a new and fast validated HPLC method that has shown that only 38% of samples were labelled within a ±10% variance from the label claim. Similar results were found in 2017 by research scientists at the University of Pennsylvania that looked at the CBD content in 84 products from 31 companies that included oils, tinctures and vape articles. Their data showed that 43% of these products were underlabelled in terms of CBD concentration, 26% were overlabelled and only 31% were labelled within a ±10% variance. Interestingly, the most mislabelled products were the e-liquids for EC whereas oil products were the most accurate^26^. Storage stability tests on these e-liquids were also carried out to investigate the effects of two factors, temperature and light, on the quality of these products upon storage. The results, the first of this kind to be gained, have shown that storing e-liquids at 4 °C allows a minimal to none degradation of CBD over a period of 30 days preserving the quality of these products. Similar results were observed when the e-liquids were stored at RT in absence of light suggesting that if clear indications are provided on the best storage conditions and light-protective containers are used, any detrimental effect can be avoided or minimized. Hence, at the present, one of the major obstacles to make electronic cigarettes a viable alternative to oral administration of CBD is represented by the inaccuracy of manufacturers and the lack of clear storing conditions on labels. Considering that, hemp-derived CBD market has exceeded 500 million dollars in 2018 and it is foreseen to dramatically grow 40 times to reach 22 billion by 2022^27^, the regulatory grey zone under which products containing cannabidiol have been sold over the past few years should be firmly addressed by health departments and regulatory authorities. In particular, standardized laboratory testing for the quantification of cannabidiol, as the analytical method herein described, should be mandatory for all products to protect consumers and their health. We also believe that studies on the actual dose *per* puff of CBD in EC should be carried out to assess the exact amount of phytocannabinoid that is biologically available in the aerosol after the vaporization process. This will be fundamental for any therapeutic use of this potential delivery system and will be the subject of our future work.

## Methods

To better estimate the concentration of CBD in commercially available products, we purchased online 13 e-liquids containing CBD with different declared concentrations of phytocannobinoid (Table 1, label CBD concentration) from the following manufactures: sample A (brand name seven wonders CBD 400 by Vaporart), sample B (brand name seven wonders CBD 200 by Vapoart), sample C (brand name CBD 200 sativa Mr Kush by CBD crystal), sample D (brand name Ambrosia by Enecta), sample E (brand name mango kush by Kanalife), sample F (brand name pure base by Harmony), sample G (brand name CBD liquid 1% by C-juice), sample H (brand name CBD liquid 2,5% by C-juice), sample I (brand name CBD liquid 5% by C-juice), sample L (brand name CBD liquid 10% by C-juice), sample M (brand name CBD pure 250 by TNT vape), sample N (brand name CBD 2% by Pure) and sample O (brand name CBD sativa blend by Sensi Seed). Products of the same company but with different contents of CBD were also selected to study its accuracy (Table 1, entry Company). Each sample was stored according to packaging instructions or, if none were provided, in a cool, dry place, and analysed within two weeks from goods reception. HPLC-grade ethanol and acetonitrile were purchased from VWR International. Reagent-grade formic acid was purchased from Aldrich. An analytical standard of cannabidiol solution, used as reference standard, was purchased from Aldrich. An Agilent 1200 RRLC system equipped with a temperature controlled autosampler, binary pump, and diode array detector (Agilent Technologies, Mississauga, ON, Canada) was used to quantify CBD by liquid chromatography (LC). The quantification was achieved on a Synergi C-18, column 250 × 4.6 mm, 4 μm (Phenomenex, Torrance, CA). Mobile phase compositions were (A) 0,1% formic acid in milli-Q water and (B) 0,1% formic acid in acetonitrile using gradient conditions at 0.5 mL/min. The separation was achieved according to the following gradient: 0–10 min, 20–90%B; 10–18 min, 90%B; 18–21 min, 90–20%B; 21–29 min, 90%B. The injection volume was 10 μL and detection was at 220 nm. The autosampler was maintained at 4 °C. Serial dilutions with ethanol of a cannabidiol solution (Aldrich analytical standard) were used to prepare five-point standard calibration curve in concentrations ranging from 5 to 500 μg/mL that were analysed in triplicate. A coefficient of determination (R2) of 0.995 was used for quantitation. Accuracy was measured by the method of standard addition. The developed method was used to determine the cannabinoid content before and after spiking. The ratio of measured to added amount was used to calculate recovery. The recovery of CBD was calculated to be>90% and <120%, indicating that the method was accurate and reliable. The limits of detection (LOD) and quantitation (LOQ) were calculated based on the standard deviation of the y-intercept (σ) and slope of the calibration curve (S) obtained from linear regression. LOD was calculated using the expression 3.3 σ/S and LOQ was calculated using 10 σ/S obtaining 1.70 and 5.00 μg/mL respectively. Thermal stability tests were carried out by transferring 1 mL of each commercially available e-liquid into an Eppendorf wrapped with aluminium foil and then left in a fridge (4 °C), in the laboratory (RT = 22 °C) and in an incubator chamber (37 °C) for 30 days. Samples were then diluted with ethanol and analysed by LC using the previously described method. Photostability tests were carried out by transferring 1 mL of each commercially available e-liquid into two distinct Eppendorf, one wrapped in aluminium foil (dark control), and exposed alongside to natural day light of a window (March 2018) for 30 days. Samples were then accordingly diluted with ethanol and analysed by LC using the previously described method. Statistical analyses were performed considering the differences in CBD concentration (expressed in mg/ml) as response variable. A Linear Mixed Effect Model (LME) was applied to evaluate the effect of temperature and light storage condition on CBD conservation. Samples were considered as random effect and residuals from the model were tested for their normality and homoschedasticity. To evaluate the significance of the loss of CBD in each sample among the three temperature storage conditions, one-way ANOVA followed by a Tukey-Kramer test for multiple comparisons was applied. To evaluate the effect of light storage conditions on the loss of CBD in each sample a *t*-test was performed. The threshold of statistical significance was set at 0.05 (*p* < 0.05).
