# Author Correction: Quantification of the content of cannabidiol in commercially available e-liquids and studies on their thermal and photo-stability

**DOI:** 10.1038/s41598-021-96039-7

**Published:** 2021-08-10

**Authors:** Carlo Mazzetti, Emanuele Ferri, Monica Pozzi, Massimo Labra

**Affiliations:** 1 Department of Biotechnology and Biosciences, University of Milano-Bicocca, Piazza della Scienza 2, 20126 Milano, Italy; 2 TRUSTICERT SRL, via Mazzini 18/C, 22036 Erba, Italy

Correction to: *Scientific Reports* 10.1038/s41598-020-60477-6, published online 28 February 2020

The original version of the Article contained an error in the title of the paper, where the word “cannabidiol” was incorrectly given as “cannabinol”.

In addition, Figure 1 contained an error in the chemical structure for “Cannabidiol (CBD)”, where H_3_C was erroneously repeated and the double bond connecting H_3_C to HO was omitted.

The original Figure 1 and accompanying legend appear below.

**Figure 1:**
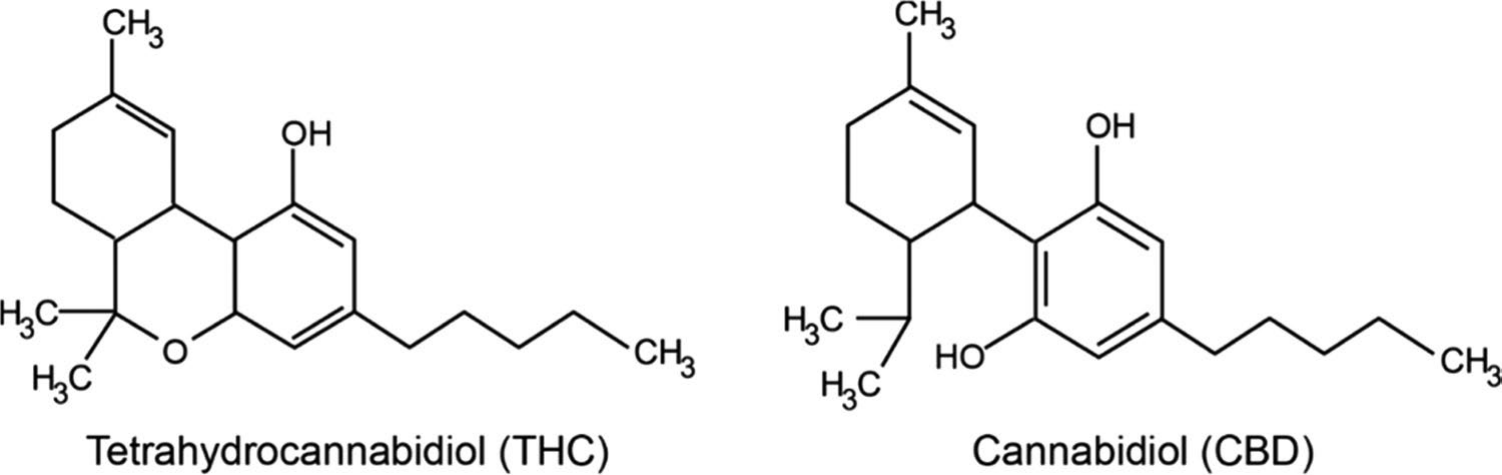
Chemical structures of THC and CBD.

The original Article has been corrected.

